# ﻿Holotype sequencing of *Silvataresholzenthali* Rázuri-Gonzales, Ngera & Pauls, 2022 (Trichoptera, Pisuliidae)

**DOI:** 10.3897/zookeys.1159.98439

**Published:** 2023-04-24

**Authors:** Jacqueline Heckenhauer, Ernesto Razuri-Gonzales, Francois Ngera Mwangi, Julio Schneider, Steffen U. Pauls

**Affiliations:** 1 Senckenberg Research Institute and Natural History Museum Frankfurt, Frankfurt, Germany Senckenberg Research Institute and Natural History Museum Frankfurt Frankfurt Germany; 2 LOEWE Centre for Translational Biodiversity Genomics (LOEWE‐TBG), Frankfurt, Germany LOEWE Centre for Translational Biodiversity Genomics Frankfurt Germany; 3 Centre de Recherche en Sciences Naturelles, Lwiro, Bukavu, Democratic Republic of the Congo Centre de Recherche en Sciences Naturelles Bukavu Democratic Republic of the Congo; 4 Institute for Insect Biotechnology, Justus-Liebig-University, Gießen, Germany Justus-Liebig-University Gießen Germany

**Keywords:** Caddisflies, extended specimen, holotype genomics, taxonomy

## Abstract

While DNA barcodes are increasingly provided in descriptions of new species, the whole mitochondrial and nuclear genomes are still rarely included. This is unfortunate because whole genome sequencing of holotypes allows perpetual genetic characterization of the most representative specimen for a given species. Thus, *de novo* genomes are invaluable additional diagnostic characters in species descriptions, provided the structural integrity of the holotype specimens remains intact. Here, we used a minimally invasive method to extract DNA of the type specimen of the recently described caddisfly species *Silvataresholzenthali* Rázuri-Gonzales, Ngera & Pauls, 2022 (Trichoptera: Pisuliidae) from the Democratic Republic of the Congo. A low-cost next generation sequencing strategy was used to generate the complete mitochondrial and draft nuclear genome of the holotype. The data in its current form is an important extension to the morphological species description and valuable for phylogenomic studies.

## ﻿Introduction

In zoology, especially when considering invertebrates, new species are often not recognized as such in the field due to the minute size of the structures used to differentiate them from already described species. Intensive treatment (e.g., preparation and preservation) and careful examination of the collected specimens are required to determine if they are indeed undescribed. In addition, many new species are discovered in regions of the world where the scientific infrastructure is insufficient to guarantee high-quality, unfragmented DNA in collected specimens. Such was also the case for the holotype of *Silvataresholzenthali* Rázuri-Gonzales, Ngera & Pauls, 2022 (Trichoptera: Pisuliidae) (Rázuri-Gonzales et al. 2022). This species belongs to the African endemic family Pisuliidae. Currently, there are 12 valid species in the genus. The taxonomic history and distribution of *Silvatares* were described by Stoltze (1989), discussed in detail by Prather and Holzenthal (2002), and most recently summarized by Rázuri-Gonzales et al. (2022).

The holotype specimen (SMFTRI00018633) was collected by FNM in the eastern D.R. Congo in 2017 and preserved in locally produced 80% ethanol. By the time the specimen was identified as representing a new species, it had been transferred into new ethanol, analyzed multiple times under the stereoscope, and shipped between countries. Without the possibility of cooling the preservative or the specimen in the D.R. Congo, it was clear that the DNA of this specimen would be substandard to what might be extractable from a freshly caught caddisfly specimen preserved in high-quality ethanol and with uninterrupted cooling. However, the described scenario for the holotype of *S.holzenthali* is the norm rather than the exception. In this paper, we want to showcase that it is possible and very valuable to generate a genomic resource from holotypes, even if the quality of the starting DNA is far from ideal.

Many initiatives are currently trying to harness recent technological developments to sequence and produce reference genomes for all species on Earth (Lewin et al. 2018; Rhie et al. 2021; Blaxter et al. 2022; Formenti et al. 2022). A reference genome is a highly contiguous, accurate, and annotated genome assembly, which represents the structure and organization of the genome of a species at a particular point in time (Formenti et al. 2022). These endeavors are crucial for documenting the Earth’s biodiversity at its most fundamental organization level (i.e., genomic diversity). Understandably, these initiatives focus first on those species that are relatively easy to sequence (i.e., often larger species where tissue is available without destroying the entire specimen and where targeted sampling of freshly collected tissues, cells, or specimens is possible). Attempts to sequence the genome of even the tiniest individuals with minimal input DNA are becoming possible (Schneider et al. 2021), but they still cannot reach the quality standards required for reference genome assemblies. The same is true for specimens and holotypes collected in scenarios similar to the one described above for *S.holzenthali*.

Another limitation of many genome sequencing initiatives is that they generally do not focus on the holotype of a species. However, in the currently accepted type-based taxonomy, the holotype (or, if necessary, the designated lectotype and neotype) serves as a species’ reference. For many species, sequencing a reference genome from the holotype is not a viable option. Many type specimens are old, and naturally, all type specimens are rare and of singular value, requiring special care, and non-invasive DNA extraction methods for genome sequencing. Thus, reference genome sequencing initiatives that require ample amounts of high-quality DNA for long-read sequencing technologies are logically and correctly focused on less valuable specimens, at best, from the *locus typicus* or from a paratype. Nevertheless, sequencing the holotype of a species allows for the genetic characterization of the most representative specimen for a given species as an eternal digital reference. Here we show that using a minimally invasive method to extract DNA from poorly preserved specimens allows taxonomists to capture and present the genetic characterization of the holotype while maintaining most of its morphological and structural integrity.

## ﻿Materials and methods

### ﻿DNA extraction, library preparation, whole genome sequencing, and sequence read processing

**Figure 1.**
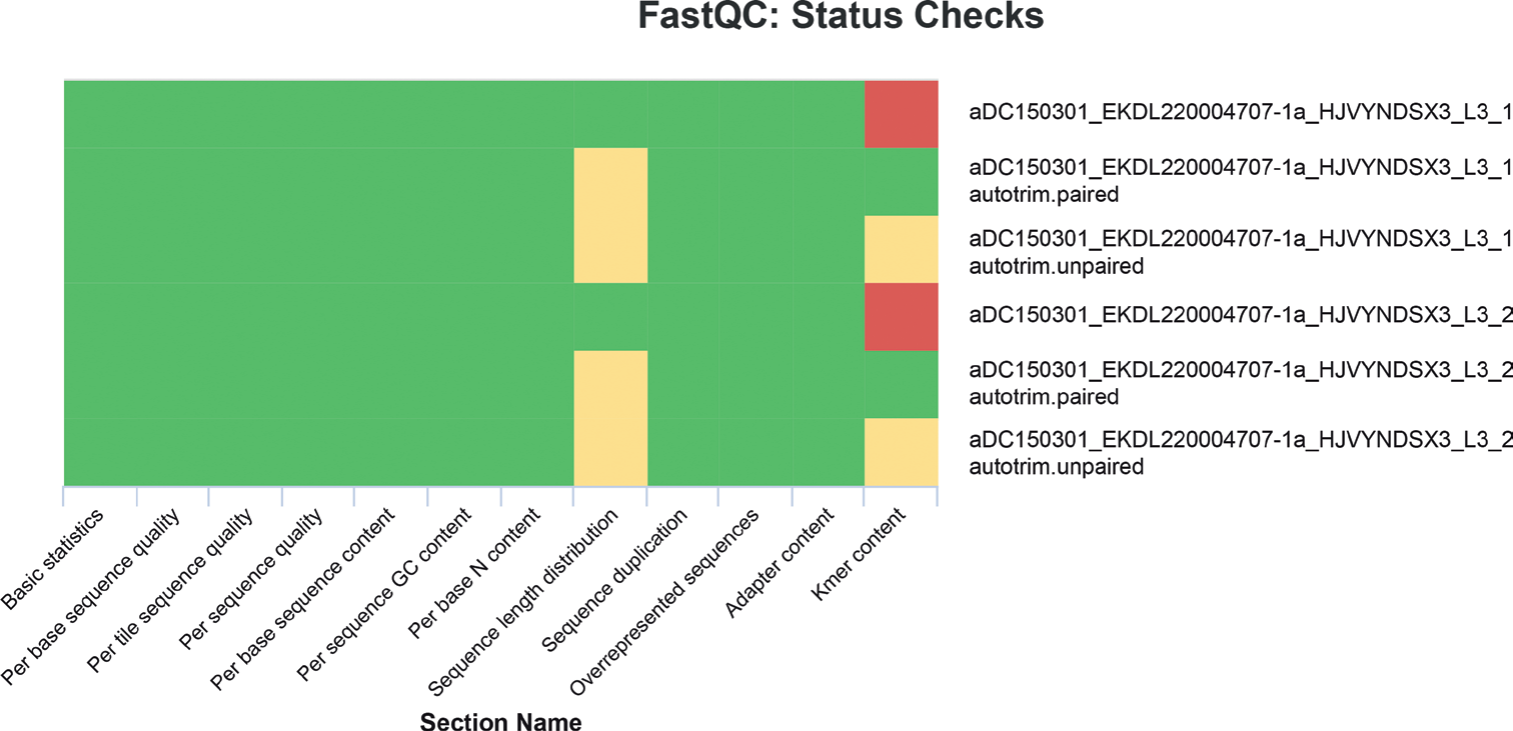
FastQC status checks of raw and trimmed reads (*autotrim), green: good, yellow: ok, red: failed.

Genomic DNA was extracted from two legs as described in Rázuri-Gonzales et al. (2022). A total of 110 ng gDNA was sheared to a mean fragment size of about 420 bp using a Bioruptor Pico (Diagenode, Seraing, Belgium). Genomic libraries were prepared using the NEBNext Ultra II DNA Library Preparation Kit for Illumina (New England Biolabs, Ipswich, MA, USA) according to the manufacturer’s manual. Adapters were diluted 1:10 as recommended for low input libraries, and size selection was conducted based on the insert size using SPRIselect beads (Beckman, Indianapolis, USA). A dual indexing PCR was run for eight cycles on a Mastercycler (Eppendorf, Germany). After cleanup, the library was eluted in 0.1X TE and shipped for 150 bp paired-end sequencing (ordering 20 Gb output) on a partial lane of an Illumina NovaSeq 6000 platform (San Diego, CA) at Novogene (Cambridge, UK). Raw reads are deposited at the NCBI SRA archive under the accession number SRR22404850. The quality of the raw reads was evaluated using FastQC v.0.11.9 (Andrews 2019). FastQC reports were summarized with MultiQC v.0.10 (Ewels et al. 2016, Fig. 1). Raw reads were trimmed for low-quality regions, adapter sequences, and over-represented *k-mers* using autotrim.pl v.0.6.1 (Waldvogel et al. 2018) and Trimmomatic v.0.39 (Bolger et al. 2014) using the adapter_all.fa of Trimmomatic and the following settings ILLUMINACLIP:2:30:10:8:true, SLIDINGWINDOW:4:20, MINLEN:50, and TOPHRED33 (Fig. 1). Unpaired reads were discarded. Contaminated reads were filtered using Kraken v.2.0.9 (Wood and Salzberg 2014). The quality of trimmed, contamination-free reads was evaluated with FastQC as described above.

### ﻿Genome size estimation and genomic characterization

We used two different approaches to estimate the genome size. First, we used a k-mer distribution-based method. For this, *k-mers* were counted with JELLYFISH v.2.3.0 (Marçais and Kingsford 2011) using jellyfish count -C -s 1000000000 -F 2 and a k-mer length of 21 based on the raw sequence reads. A histogram of *k-mer* frequencies was created with jellyfish histo and used for analysis with the online web tool GenomeScope v.2.0 (Ranallo-Benavidez et al. 2020) using the following parameters: *k-mer* length = 21, ploidy = 2, max *k-mer* coverage = 10000. In addition, we estimated genome size with a re-mapping-based approach using backmap.pl (Schell et al. 2017; Pfenninger et al. 2022). This wrapper script uses the following dependencies samtools (Li et al. 2009), bwa mem (Li 2013), qualimap (Okonechnikov et al. 2015), MultiQC (Ewels et al. 2016), bedtools (Quinlan and Hall 2010), and RScript (R Core Team 2021) to automatically map the trimmed, contamination-free reads to the assembly (see *de novo* nuclear genome assembly) with bwa mem. Then, it executes qualimap bamqc and finally estimates genome size by dividing the mapped nucleotides by the mode of the coverage distribution (>0).

### ﻿Mitogenome assembly

The mitochondrial genome was first assembled with the raw reads using NOVOplasty v.4.2 (Dierckxsens et al. 2016) using the following parameters: type = mito, genome range = 12000–22000, *k-mer* = 33, max memory = 100, read length = 150, insert size = 300, platform = illumina, paired = PE, insert size auto = yes. The partial sequence of the *cytochrome c oxidase subunit I* (*COX1*) gene of *Silvataresensifera* Barnard, 1934, KX291165, was used as seed input. All other parameters were kept as default. The circularized mitogenome was aligned to the complete mitochondrial sequence of *Phryganeacinerea* Walker, 1852, MG980616, with MAFFT in Geneious Prime v.2022.1.1 with default settings to set the correct start position. Annotation of tRNAs, rRNAs, and protein-coding genes was done with MitoZ v.2.3 (Meng et al. 2019) using the module “annotate with genetic_code 5” and clade Arthropoda. Positions of *trnL*, *trnT*, and *trnS* were manually curated based on the alignment to *P.cinerea*. The mitochondrial genome assembly was deposited in GenBank under the accession OP921089.

### ﻿*De novo* nuclear genome assembly

Nuclear genome assembly was conducted in Spades v.3.14.1 (Bankevich et al. 2012) with the default settings. After scaffolds smaller than 500 bp and those matching the mitochondrial genome assembly were filtered out, assembly statistics were calculated with Quast v.5.0.2 (Gurevich et al. 2013), and quality was assessed in several ways. First, completeness was accessed via screening for single-copy orthologs with BUSCO v.4.1.4 (Simão et al. 2015) using the endopterygota_odb10 dataset. Second, the backmapping rate of the trimmed reads to the assembly was calculated with backmap.pl 0.3 as described above (see “Genome size estimation and genomic characterization”). Third, the final genome assemblies were screened for potential contaminations with taxon-annotated GC-coverage (TAGC) plots using BlobTools v.1.1.1 (Laetsch and Blaxter 2017). For this purpose, the bam file resulting from the backmapping analysis was converted to a blobtools readable cov file with blobtools map2cov. Taxonomic assignment for BlobTools was done with blastn 2.10.0+ (Camacho et al. 2009) using -task megablast and -e-value 1e-25. The blobDB was created and plotted from the cov file and blast hits. The nuclear draft genome assembly was deposited in GenBank under the accession JAPMAF000000000. All commands used in this study are given in Suppl. material 1.

## ﻿Results

### ﻿Whole genome sequencing and genome characterization

Illumina sequencing resulted in 160 534 832 raw short reads with a data amount of 24.1 Gb. 3.3% of reads were identified as contaminated (2.7% *Homosapiens*, 0.6% bacteria, 0.1% viruses, 0.03% other). Over-represented *k-mers* were successfully removed using autotrim.pl v.0.6.1 (Fig. 1). After trimming and contamination filtering, 149 928 720 reads (~21.8 Gb) were kept.

**Figure 2.**
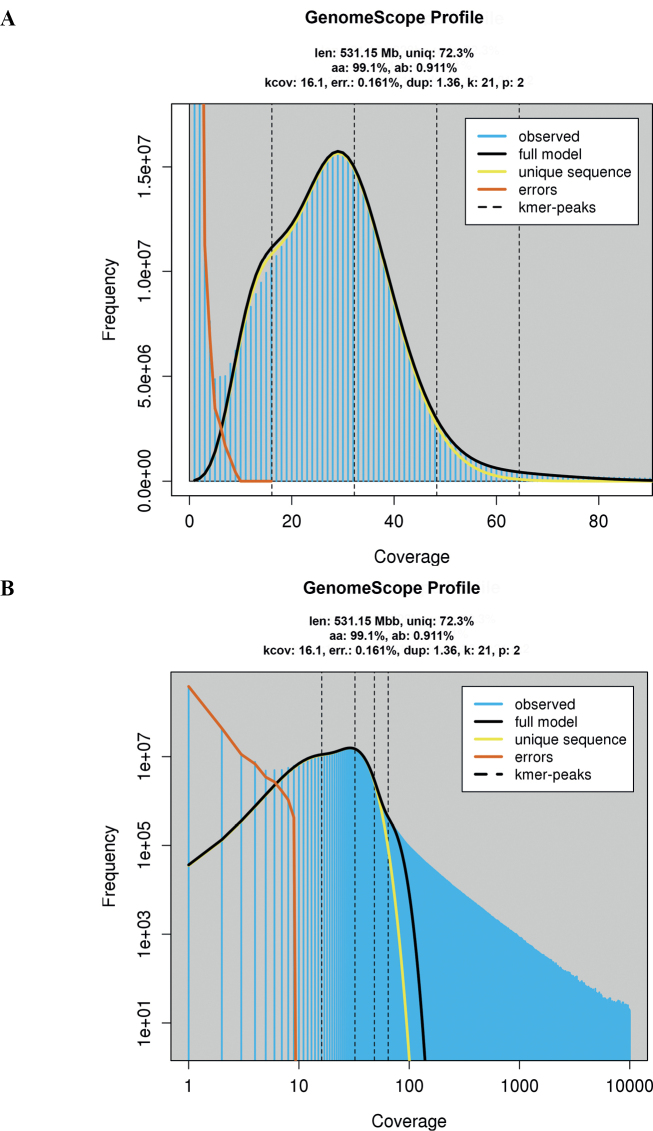
Genomescope2 profiles **A** linear plot **B** log plot; len: inferred total genome length, uniq: percent of the genome that is unique (not repetitive), kcov: mean *k*-*mer* coverage for heterozygous bases, err: error rate of the reads, dup: average rate of read duplications.

**Figure 3.**
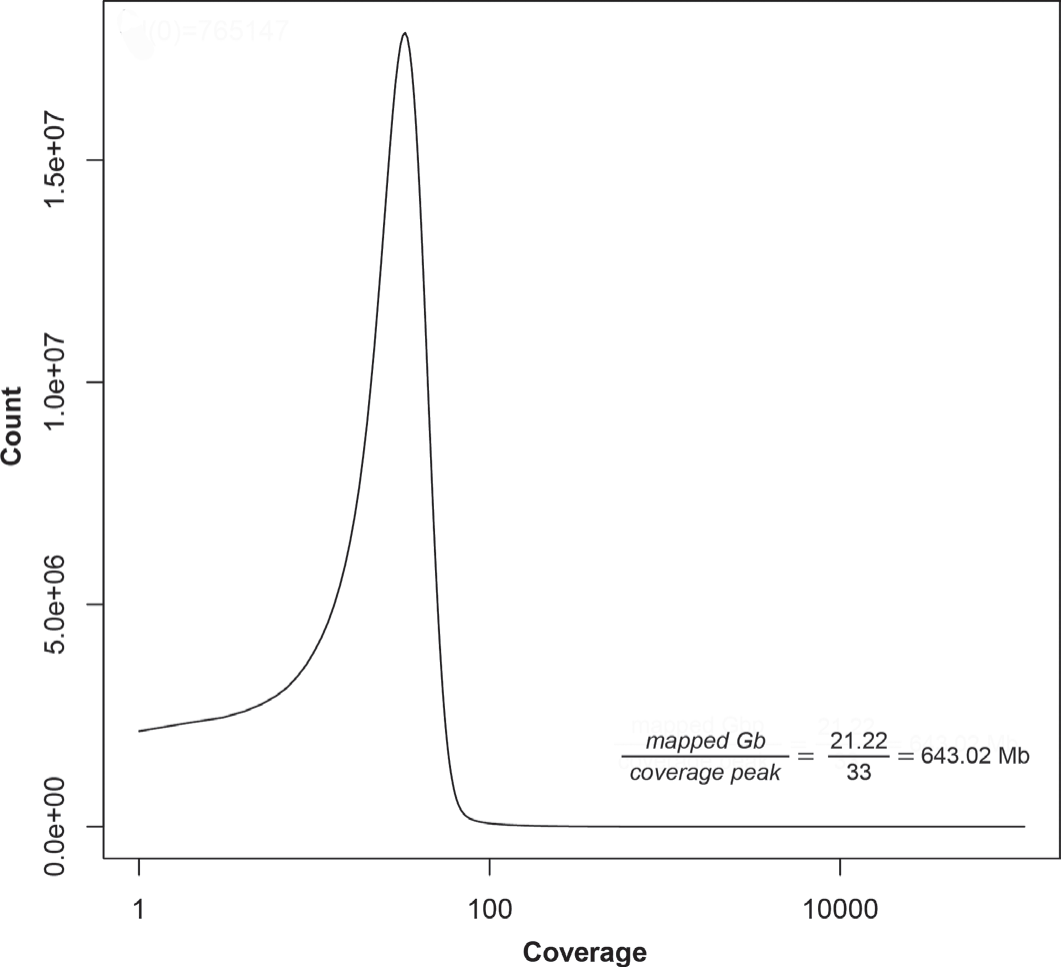
Coverage distribution per position. The x-axis is given in log-scale. Mapped nucleotides: 21.22 Gb. The peak coverage is 33. This results in genome size estimation of 643.02 Mb.

*K-mer* analysis based on raw read data estimated the genome size to be 531.15 Mb, with a heterozygosity of 37.7% (Fig. 2), while backmap.pl revealed a genome size of 643.02 Mb (Fig. 3).

### ﻿Mitochondrial genome

**Figure 4.**
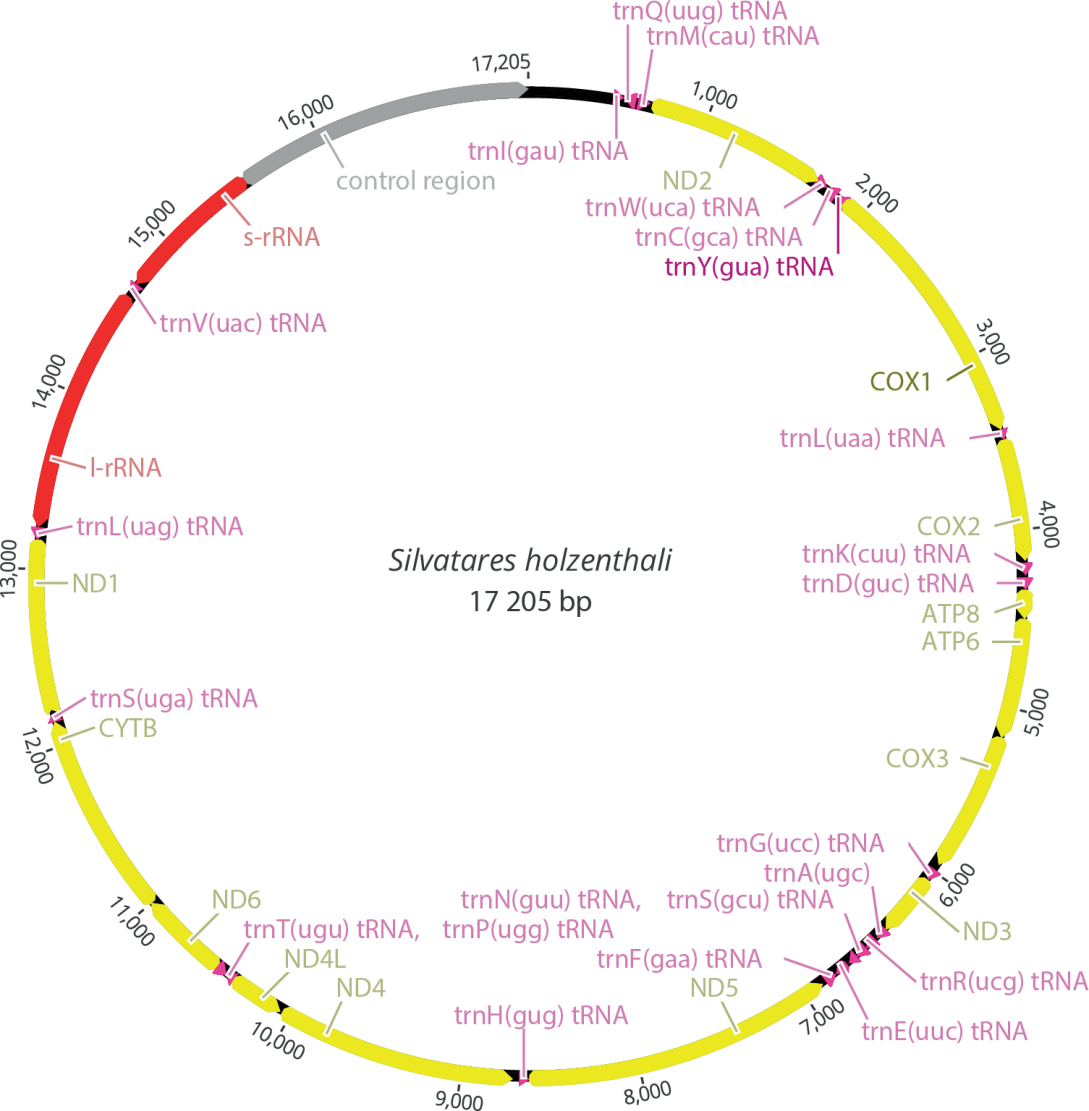
Circular mitochondrial genome of the holotype of *Silvataresholzenthali*.

The NOVOplasty assembly resulted in a 17 205 bp-long and circularized contig (Fig. 4). Its annotation revealed all expected 13 protein-coding genes and both rRNAs and 23 tRNAs. The d-loop was manually curated based on a comparison with the complete mitochondrial sequence of *Limnephilusdecipiens* Kolenati, 1848, AB971912.

Standard abbreviations are given for protein-coding (yellow), transfer (pink), and ribosomal RNA (red) genes. The control region is shown in gray. Orientation of genes is indicated by direction of arrows.

### ﻿Nuclear genome

**Figure 5.**
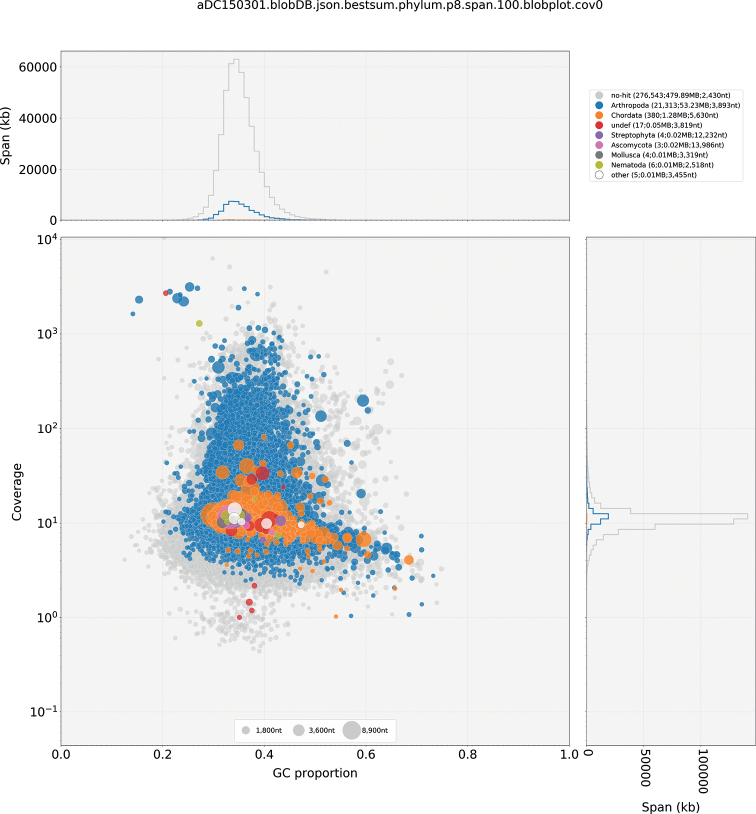
Taxon-annotated GC-coverage (TAGC) plots for the nuclear genome assembly. Scaffolds are represented with circles. Colors indicate the best match to the corresponding taxonomic annotation (grey= no hits, blue= Arthropoda, for other colors see legend in the figure, upper right box). The distribution of the total span (kb) of contigs for a given GC proportion or coverage is given in the upper- and right panels, respectively.

The nuclear genome assembly contains 298 265 scaffolds with a total length of 534.50 Mb, an N50 of 2 549, and a GC of 35.27%. 99.07% of reads were mapped back to the assembly. The BUSCO search with 2 124 Endopterygota orthologs resulted in 74.7% BUSCOs; of these, 44.7% were complete (44.3% single, 0.4% duplicated), and 31% were fragmented. Blobtools detected no contaminations based on GC content and coverage distribution (Fig. 5). While uploading the genome to NCBI, NCBI’s contamination screening detected and filtered a 29 bp-long contamination (vector, etc.) at the beginning of one scaffold.

## ﻿Discussion

While the morphology of the genus *Silvatares* has been described extensively, less than a handful of partial genes have been published or uploaded to NCBI GenBank. For example, *cadherin*, *cytochrome oxidase subunit 1* (*COX1*), and the *28S* large subunit and *18S* small subunit ribosomal RNA are available for *S.ensifera* (MN364796, KX291165, KX106901, AF436522, AF436172, AF436293, MN296628, AF436410); *carbamoylphosphate synthase domain protein*, *isocitrate dehydrogenase*, *RNA polymerase II*, and *COX1* for *Silvatares* sp. (KC559510, KC559654, KC559734, KC559575); *COX1* for *S.collyrifer* Barnard, 1934, (KX291056); and *COX1* for *S.thrymmifer* Barnard, 1934 (MN344469; MN344493) (Malm et al. 2013; Zhou et al. 2016; Thomas et al. 2020).

Here, we present the mitogenome and a draft nuclear genome assembly through our ~45× sequencing coverage of short-read data. This genome assembly is admittedly far away from the quality standards of a reference genome; however, we argue that this genomic resource is still an invaluable addition to the characterization of the holotype of *Silvataresholzenthali*. The genome assembly reported in this study includes all the partial genes that had been hitherto sequenced for other *Silvatares* species, as well as 74.7% of the 2 442 benchmarking universal single-copy orthologs in Endopterygota. Additionally, this assembly provides the complete mitogenome, including the barcode markers. This highlights that for a few hundred dollars we can produce much more genomic information on type specimens than the “DNA barcode,” which has already become an important addition to many morphological species descriptions (e.g., Hebert and Gregory 2005; Padial and De la Riva 2007; Pohl et al. 2012; Egan et al. 2017). Sequencing the genome of the holotype permanently links the genetic characterization to the name-bearing specimen of a given species. This information is very valuable for studying the systematics and evolution of the species in question. Especially in variable taxa or clades with high levels of cryptic diversity, anchoring species delimitation analyses, taxonomic work or evolutionary studies on the genetic make-up of the name-bearing specimens can be extremely helpful. Genome wide data have been used to help delimit closely related species in Trichoptera (e.g., Deng et al. 2021); however, since the holotypes of the species in question were not among the analyzed specimens, the nomenclature and taxonomy of each species could not be fully resolved. This case highlights the value of sequencing the genome of the primary type. Since the genome of *S.holzenthali* is the first holotype genome in caddisflies there are no examples of species delimitation based on the name-bearing type specimen yet.

In other taxa, holotype genomes have already been published. In a pioneering study, Pohl et al. (2012) provided a complete short-read-based genome in the description of a new Strepsiptera species using specimens from the type series. Since then, *de novo* genomes are increasingly included in new species descriptions across the animal kingdom, such as in *Caenorhabditis* Osche, 1952 (Kanzaki et al. 2018), mud snakes and frogs (Köhler et al. 2021a, b, c), gall wasps (Brandão-Dias et al. 2022), fungi (Emanuel et al. 2022) and fishes (Sullivan et al. 2022).

The draft genome assembly generated in this study can be applied in population genetic studies, for example, to assess the heterozygosity of the type specimen as a proxy for population genetic variation at the time of sampling (Köhler et al. 2021a). Furthermore, the data is valuable in a phylogenomic context (Brandão-Dias et al. 2022). For other downstream genomic analyses, the provided data from the holotype can always be mapped to a higher-quality reference genome generated from specimens of lesser value and better DNA quality. Notably, the approach we present here also lends itself to museum specimens, which are usually of older age. Being able to tap into these immense and often irreplaceable resources for genomic study opens a wealth of scientific opportunity and has developed in the growing genomic field of museomics (Raxworthy and Smith 2021) which propagates generating genomic data from historical specimens using a variety of methods. This includes shotgun genome sequencing as presented here, but also hybrid capture approaches for degraded DNA once appropriate bait sets have been developed (Bi et al. 2013; Raxworthy and Smith 2021; Castañeda-Rico et al. 2022).

While we think a *de novo* genome or a genomic resource of any kind is an invaluable added resource and important additional diagnostic character in species descriptions, priority should be given to preserving specimen integrity of the type specimens. Most methods for extracting DNA ultimately cause at least minimal structural damage to the holotype. In our case, this damage (removing and clearing the abdomen; DNA extraction from two legs) was necessary to recognize and identify the holotype as a new species. No additional damage was done to extract DNA for generating the genome. However, in other situations, the methods used to preserve and store insects may not always allow for generating a *de novo* genome from holotypes without causing significant additional damage to the type. In this case we maintain that priority should be given to safekeeping the structural integrity of the holotype specimen, and genomic information possibly obtained from a paratype or a duplicated structure on the holotype.

## Supplementary materials

Supplementary material 1 Commands used in the study Heckenhauer, J., Rázuri-Gonzales, E., Mwangi, F.N., Schneider, J., Pauls, S. U. (2022) Holotype sequencing of *Silvataresholzenthali* (Trichoptera: Pisuliidae) Data type Bioinformatic commands https://binary.pensoft.net/file/841902This dataset is made available under the Open Database License (http://opendatacommons.org/licenses/odbl/1.0/). The Open Database License (ODbL) is a license agreement intended to allow users to freely share, modify, and use this Dataset while maintaining this same freedom for others, provided that the original source and author(s) are credited.Jacqueline Heckenhauer, Ernesto Razuri-Gonzales, Francois Ngera Mwangi, Julio Schneider, Steffen U. Pauls

Supplementary material 2 Genomic DNA degradation assessment on a TapeStation 2200 Data type DNA Extraction quality control https://binary.pensoft.net/file/841903This dataset is made available under the Open Database License (http://opendatacommons.org/licenses/odbl/1.0/). The Open Database License (ODbL) is a license agreement intended to allow users to freely share, modify, and use this Dataset while maintaining this same freedom for others, provided that the original source and author(s) are credited.Jacqueline Heckenhauer, Ernesto Razuri-Gonzales, Francois Ngera Mwangi, Julio Schneider, Steffen U. Pauls
