# Supplementary material for: ﻿Holotype sequencing of Silvataresholzenthali Rázuri-Gonzales, Ngera & Pauls, 2022 (Trichoptera, Pisuliidae)
Source: Zookeys. 2023 Apr 24;1159:1–15. doi: 10.3897/zookeys.1159.98439 (PMC10193998; doi:10.3897/zookeys.1159.98439)
Supplement: Supplementary material 1 — Commands used in the study Heckenhauer, J., Rázuri-Gonzales, E., Mwangi, F.N., Schneider, J., Pauls, S. U. (2022) Holotype sequencing of Silvataresholzenthali (Trichoptera: Pisuliidae) [file zookeys-1159-001_article-98439__-s001.pdf]

Supplementary material #1

Commands used in the study Heckenhauer, J., Rázuri-Gonzales, E., Mwangi, F.N., Schneider, J., Pauls, S. U. (2022) Holotype sequencing of Silvatares holzenthali (Trichoptera: Pisuliidae).

1. Fastqc to evaluate quality of raw reads

```
#!/bin/bash
#SBATCH --partition=mem
#SBATCH --cpus-per-task=32
#SBATCH --mem=40G
module load fastqc/0.11.9 multiqc/1.10
fastqc -t 32 aDC150301_EKDL220004707-1a_HJVYNDSX3_L3_1.fq.gz
aDC150301_EKDL220004707-1a_HJVYNDSX3_L3_2.fq.gz &&
multiqc .
```

2. Trim reads

```
#!/bin/bash
#SBATCH --partition=cpu
#SBATCH --cpus-per-task=94
#SBATCH --mem=236G
module load autotrim 0.6.1
autotrim.pl -fofn samples.fofn -trim trimmer.txt -log ./ -tt 94
```

```
cat trimmer.txt
ILLUMINACLIP:/Trimmomatic/Trimmomatic-0.39/adapters/adapter_all.fa:2:30:10:8:true
SLIDINGWINDOW:4:20 MINLEN:50 TOPHRED33

cat samples.fofn
./ aDC150301_EKDL220004707-1a_HJVYNDSX3_L3_1.fq.gz
./ aDC150301_EKDL220004707-1a_HJVYNDSX3_L3_2.fq.gz
```

3. Fastqc to evaluate quality of trimmed reads

```
#!/bin/bash
#SBATCH --partition=mem
#SBATCH --cpus-per-task=32
#SBATCH --mem=40G
```

```
module load fastqc/0.11.9 multiqc/1.10
fastqc -t 32 aDC150301_EKDL220004707-1a_HJVYNDSX3_L3_1_autotrim.paired.fq
aDC150301_EKDL220004707-1a_HJVYNDSX3_L3_2_autotrim.paired.fq
aDC150301_EKDL220004707-1a_HJVYNDSX3_L3_1_autotrim.unpaired.fq
aDC150301_EKDL220004707-1a_HJVYNDSX3_L3_2_autotrim.unpaired.fq
aDC150301_EKDL220004707-1a_HJVYNDSX3_L3_1.fq.gz aDC150301_EKDL220004707-
1a_HJVYNDSX3_L3_2.fq.gz && multiqc .
```

4. Genome size estimation: Jellyfish

```
#!/bin/bash
#SBATCH --partition=cpu
#SBATCH --cpus-per-task=64
#SBATCH --mem=995G
module load jellyfish/2.3.0
jellyfish count -F 2 -C -m 21 -s 1000000000 -t 64 < (zcat aDC150301_EKDL220004707-
1a_HJVYNDSX3_L3_1.fq.gz aDC150301_EKDL220004707-1a_HJVYNDSX3_L3_2.fq.gz) -o
aDC150301.jf && jellyfish histo -t 64 aDC150301.jf > aDC150301.histo && rm
aDC150301.jf
```

5. Mitogenome assembly

```
#!/bin/bash
#SBATCH --partition=cpu
#SBATCH --cpus-per-task=1
#SBATCH --mem=100G
module load novoplasty/4.2
create_config.sh > config.txt
NOVOPlasty4.2.pl -c config.txt
```

6. Annotation of the mitogenome assembly obtained with Mitoz

The mitogenome was aligned to Genbank Accession AB971912 in Geneious Prime 2022.1.1 to set start of the mitogenome.

```
udocker run --rm --volume=$PWD --workdir=$PWD guanliangmeng/mitoz:2.3 python3
/app/release_MitoZ_v2.3/MitoZ.py annotate --genetic_code 5 --clade Arthropoda --
outprefix aDC150301_mitoz --thread_number 16 --fastafile
aDC150301_mitogenome.fasta
```

7. Contamination filtering with Kraken

```
#!/bin/bash
#SBATCH --partition=cpu
#SBATCH --cpus-per-task=48
#SBATCH --mem=115G
module load kraken2/2.1.2
kraken2 --db /cluster/software/kraken2/2.0.8/db/standard_2020-03-18 --threads 48 -
-report aDC150301.paired.kraken2.report --paired --unclassified-out aDC150301-
unclass#.fq aDC150301_EKDL220004707-1a_HJVYNDSX3_L3_1_autotrim.paired.fq
aDC150301_EKDL220004707-1a_HJVYNDSX3_L3_2_autotrim.paired.fq
```

8. Nuclear de novo assembly with resulting files from step 7

```
#!/bin/bash
#SBATCH --partition=long
#SBATCH --cpus-per-task=64
#SBATCH --mem=637G
module load spades/3.14.1
spades.py -o ./spades -1 aDC150301-unclass_1.fq -2 aDC150301-unclass_2.fq -t 64 -m
637
```

9. Filter out contigs smaller than 500

```
lengthfilter.py scaffolds.fasta fasta 499 scaffolds_ aDC150301_ge500.fasta
```

10. Filter out mitochondrial contigs from nuclear genome

The mitogenome was blasted against the nuclear assembly using megablast in Geneious Prime 2022.1.1.

```
awk 'BEGIN{while((getline<"ids.txt")>0)l[">"$1]=1}/^>/{f=!l[$1]}f' scaffolds_
aDC150301_ge500.fasta > scaffolds_ aDC150301_ge500_womito.fasta
```

```
cat ids.txt
NODE_26403_length_4027_cov_16.444557
NODE_51832_length_2751_cov_3380.376215
NODE_46821_length_2933_cov_3221.739146
NODE_68092_length_2277_cov_4612.981818
NODE_99883_length_1673_cov_3260.248747
NODE_182103_length_925_cov_4482.292453
NODE_258969_length_608_cov_2655.404896
NODE_199119_length_839_cov_4211.530184
NODE_233426_length_696_cov_3704.927302
NODE_202563_length_823_cov_4550.242627
```

11. Get assembly statistics with quast

```
module load quast/5.0.2
quast.py scaffolds_ aDC150301_ge500_womito.fasta
```

12. Check for completeness with BUSCO with Endopterygota dataset

```
#!/bin/bash
#SBATCH --partition=cpu
#SBATCH --cpus-per-task=16
#SBATCH --mem=130G
module purge
module unload python3
module load busco/4.1.4
busco -i scaffolds_ aDC150301_ge500_womito.fasta -c 8 -o aDC150301_spades -m geno
-l /cluster/software/busco/datasets/odb10/endopterygota_odb10/ --long –offline
```

13. Check for contaminations using Blobtools

13.1. Taxonomic assignment with BLAST

```
#!/bin/bash
#SBATCH --partition=cpu
#SBATCH --cpus-per-task=16
#SBATCH --mem=130G
module load ncbi-blast/2.12.0
blastn -task megablast -query aDC150301_scaffolds_ge500.fasta -db
```

```
/cluster/software/blastdb/nt/nt -outfmt '6 qseqid staxids bitscore std' -
num_threads 16 -evalue 1e-25 -out aDC150301_vs_nt
```

13.2. Back-mapping of filtered, contamination-free reads to genome assembly

```
#!/bin/bash
#SBATCH --partition=mem
#SBATCH --cpus-per-task=8
#SBATCH --mem=100G
module load backmap
backmap.pl -pre aDC150301 -a aDC150301_scaffolds_ge500.fasta -p aD15031-
unclass_1.fq, aD15031-unclass_2.fq -t 16 -v
```

13.3. Blobtools map2cov

```
#!/bin/bash
#SBATCH --partition=mem
#SBATCH --cpus-per-task=16
#SBATCH --mem=65G
module load samtools blobtools
samtools index aDC150301.sort.bam
blobtools map2cov -i aDC150301_scaffolds_ge500.fasta" -b aDC150301.sort.bam
```

13.4. Create Blobplot

```
#!/bin/bash
#SBATCH --partition=mem
#SBATCH --cpus-per-task=16
#SBATCH --mem=65G
module load blobtools
blobtools create -i aDC150301_scaffolds_ge500.fasta -o aDC150301 -c
aDC150301.sort.bam.cov -t aDC150301_vs_nt &&
blobtools plot -i aDC150301.blobDB.json
```
