# Supplementary material for: ﻿Holotype sequencing of Silvataresholzenthali Rázuri-Gonzales, Ngera & Pauls, 2022 (Trichoptera, Pisuliidae)
Source: Zookeys. 2023 Apr 24;1159:1–15. doi: 10.3897/zookeys.1159.98439 (PMC10193998; doi:10.3897/zookeys.1159.98439)
Supplement: Supplementary material 2 — Genomic DNA degradation assessment on a TapeStation 2200 [file zookeys-1159-001_article-98439__-s002.pdf]

Supplementary material #2
Genomic DNA degradation assessment on a TapeStation 2200

Genomic DNA ScreenTape®

Filename: 2022-03-31-01_JS_Silvatares_aDC150301.gDNA

Gel Images

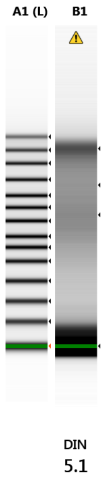

v

Default image (Contrast 50%), Image is Scaled to Sample

Sample Info

Well	DIN	Conc. [ng/µl]	Sample Description	Alert	Observations
A1	-	62.5	Ladder		Ladder
B1	5.1	4.13	aDC150301_Silvatares_spnov	⚠	Sample concentration outside functional range for DIN

 © Agilent Technologies, Inc. 2016

A1: Ladder

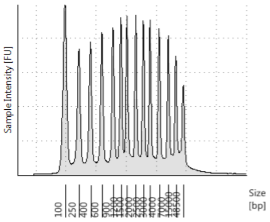

B1: aDC150301_Silvatares_spnov

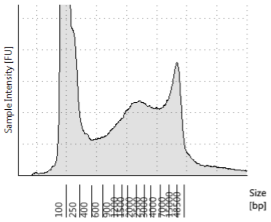

 © Agilent Technologies, Inc. 2016

A1: Ladder

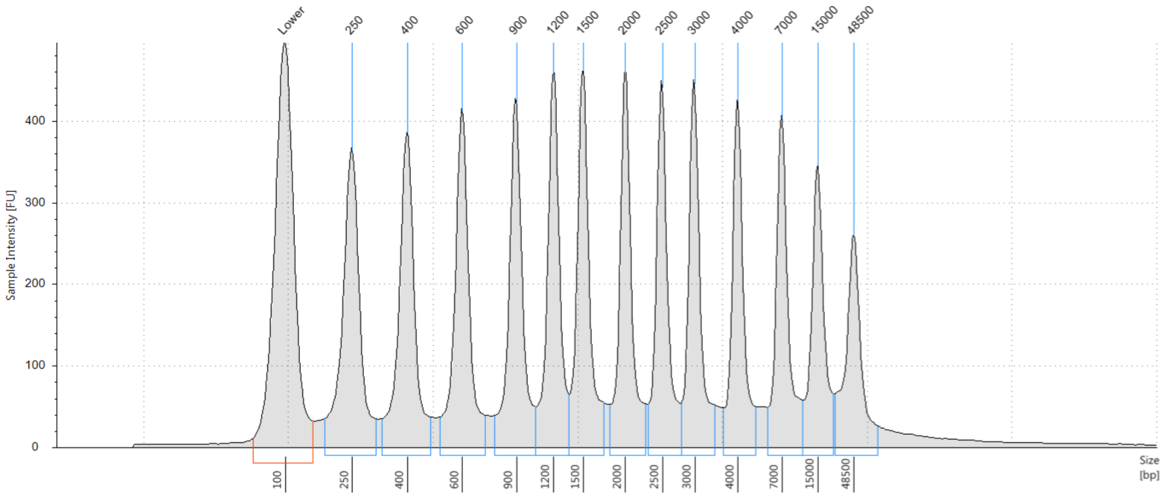

Sample Table

Well	DIN	Conc. [ng/µl]	Sample Description	Alert	Observations
A1	-	62.5	Ladder		Ladder

Peak Table

Size [bp]	Calibrated Conc. [ng/µl]	Assigned Conc. [ng/µl]	% Integrated Area	From [bp]	To [bp]	Peak Comment	Observations
100	8.50	8.50	-	64	146		Lower Marker
250	5.42	-	9.02	172	307		
400	5.17	-	8.62	323	473		
600	5.14	-	8.56	507	712		
900	4.95	-	8.25	765	1044		
1200	4.92	-	8.20	1044	1348		
1500	4.94	-	8.22	1348	1725		
2000	4.77	-	7.94	1797	2260		
2500	4.53	-	7.55	2293	2785		
3000	4.51	-	7.52	2785	3437		
4000	4.22	-	7.03	3634	5019		
7000	4.22	-	7.03	5838	10781		
15000	3.80	-	6.32	10781	20870		
48500	3.45	-	5.74	21408	>60000		

 © Agilent Technologies, Inc. 2016

B1: aDC150301_Silvatares_spnov

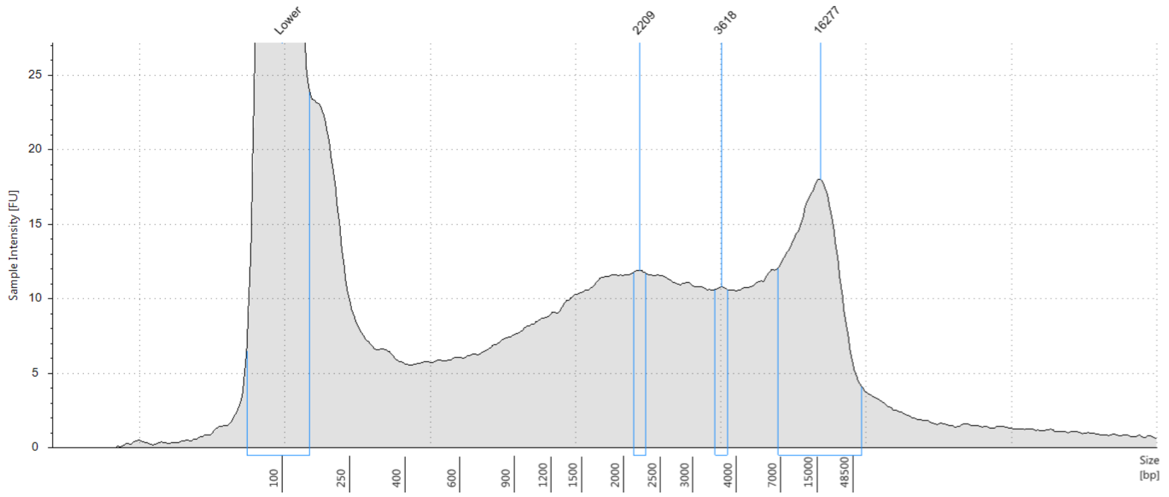

Sample Table

Well	DIN	Conc. [ng/µl]	Sample Description	Alert	Observations
B1	5.1	4.13	aDC150301_Silvatares_spnov	⚠	Sample concentration outside functional range for DIN

Peak Table

Size [bp]	Calibrated Conc. [ng/µl]	Assigned Conc. [ng/µl]	% Integrated Area	From [bp]	To [bp]	Peak Comment	Observations
100	8.50	8.50	-	62	146		Lower Marker
2209	0.111	-	10.63	2130	2291		
3618	0.0996	-	9.54	3475	3767		
16277	0.775	-	74.19	6779	56917		
-	-	-	-	-	-		Sample Well

 © Agilent Technologies, Inc. 2016

Calibration

Molecular Weight Settings

Fitting type: Genomic DNA Sizing
Alignment type: From lower marker

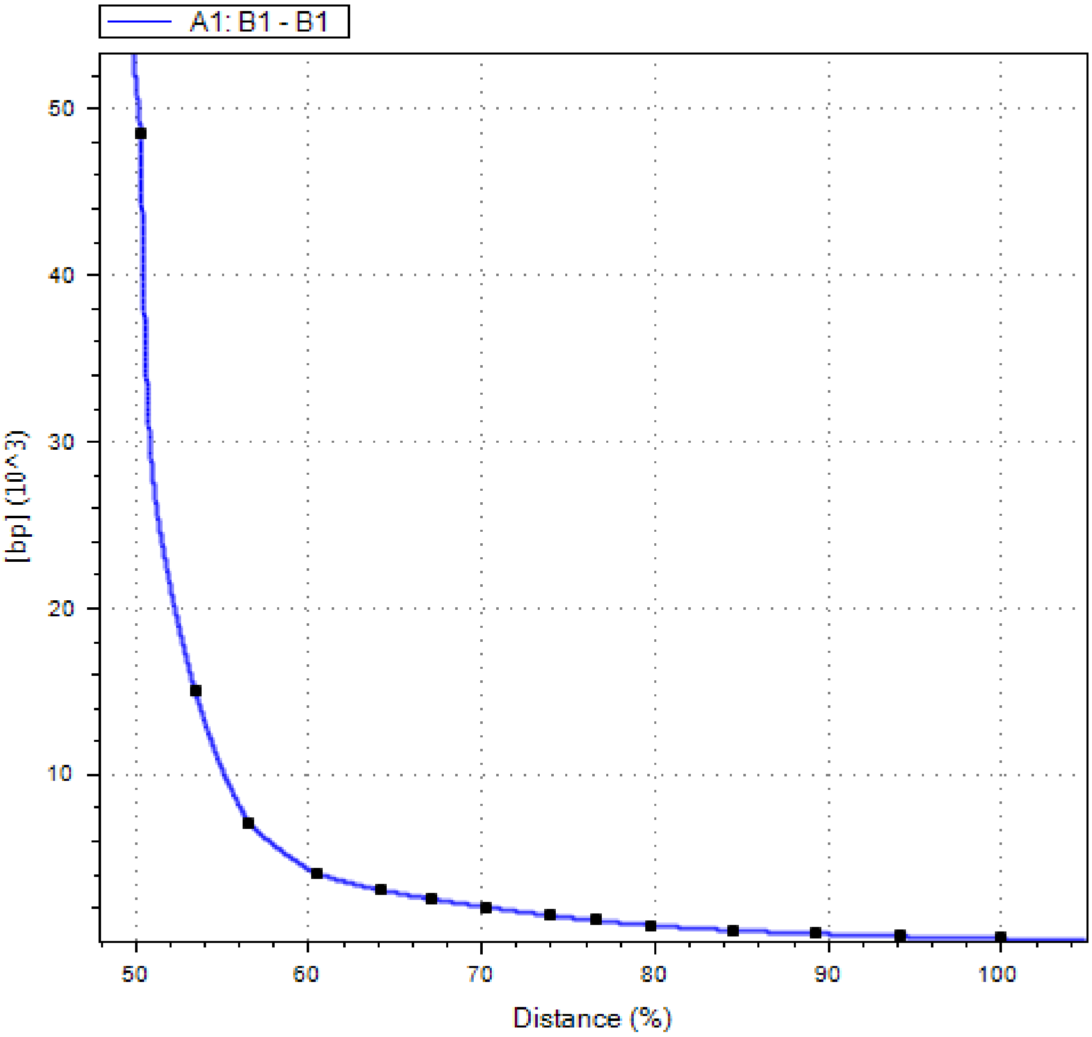

Concentration Settings

Calibration mode: Lower Marker
Normalise peaks from: Lower Marker
Fitting type: Linear Regression
